# The prognostic potential of alternative transcript isoforms across human tumors

**DOI:** 10.1186/s13073-016-0339-3

**Published:** 2016-08-17

**Authors:** Juan L. Trincado, E. Sebestyén, A. Pagés, E. Eyras

**Affiliations:** 1 Universitat Pompeu Fabra (UPF), Dr. Aiguader 88, E08003 Barcelona, Spain; 2 IFOM, the FIRC Institute of Molecular Oncology, Via Adamello 16, 20139 Milan, Italy; 3 Catalan Institution for Research and Advanced Studies (ICREA), Passeig Lluís Companys 23, E08010 Barcelona, Spain

## Abstract

**Background:**

Phenotypic changes during cancer progression are associated with alterations in gene expression, which can be exploited to build molecular signatures for tumor stage identification and prognosis. However, it is not yet known whether the relative abundance of transcript isoforms may be informative for clinical stage and survival.

**Methods:**

Using information theory and machine learning methods, we integrated RNA sequencing and clinical data from The Cancer Genome Atlas project to perform the first systematic analysis of the prognostic potential of transcript isoforms in 12 solid tumors to build new signatures for stage and prognosis. This study was also performed in breast tumors according to estrogen receptor (ER) status and melanoma tumors with proliferative and invasive phenotypes.

**Results:**

Transcript isoform signatures accurately separate early from late-stage groups and metastatic from non-metastatic tumors, and are predictive of the survival of patients with undetermined lymph node invasion or metastatic status. These signatures show similar, and sometimes better, accuracies compared with known gene expression signatures in retrospective data and are largely independent of gene expression changes. Furthermore, we show frequent transcript isoform changes in breast tumors according to ER status, and in melanoma tumors according to the invasive or proliferative phenotype, and derive accurate predictive models of stage and survival within each patient subgroup.

**Conclusions:**

Our analyses reveal new signatures based on transcript isoform abundances that characterize tumor phenotypes and their progression independently of gene expression. Transcript isoform signatures appear especially relevant to determine lymph node invasion and metastasis and may potentially contribute towards current strategies of precision cancer medicine.

**Electronic supplementary material:**

The online version of this article (doi:10.1186/s13073-016-0339-3) contains supplementary material, which is available to authorized users.

## Background

Tumors advance through stages that are generally characterized by their size and spread to lymph nodes and other parts of the body [1]. Establishing the stage of a tumor is critical to determine patient prognosis and to select the appropriate therapeutic strategy [2]. Even though stage is generally defined from a number of tests carried out on a patient, this information may sometimes be incomplete or inconclusive. Advances in the molecular characterization of tumors have led to improvements in stage classification and clinical management of patients [3]. Although tumors originate primarily from genetic lesions, their progression involves other molecular transformations, which are related to the activation of specific aggressive phenotypes, like tumor spread and metastasis, and are often reflected in gene expression changes [4, 5]. Accordingly, the development of gene expression signatures has been instrumental to complement and improve stage identification and prognosis [6–9]. On the other hand, gene expression summarizes the output of RNA transcripts from a gene locus, which is mostly explained by one transcript isoform [10]. Furthermore, we described before how solid tumors present frequent changes in the relative abundances of isoforms in comparison to normal tissues [11]. This prompts the question of whether transcript isoform changes, which remain largely unexplored as predictive signatures of tumor stage and survival, could hold relevant novel mechanisms of tumor progression. We investigated the potential of the relative abundances of transcript isoforms to determine tumor staging and clinical outcome in 12 different tumor types, integrating RNA sequencing (RNA-seq) and clinical annotation data for 12 tumor types from The Cancer Genome Atlas (TCGA) project. Our analyses revealed new signatures that characterize tumor phenotypes and their progression that are largely independent of gene expression. Knowledge about the relative abundance of transcript isoforms in tumors can potentially help predicting stage and clinical outcome and contribute towards current molecular strategies in precision cancer medicine.

## Results

### Relative abundances of transcript isoforms are predictive of tumor stage

**Fig. 1:**
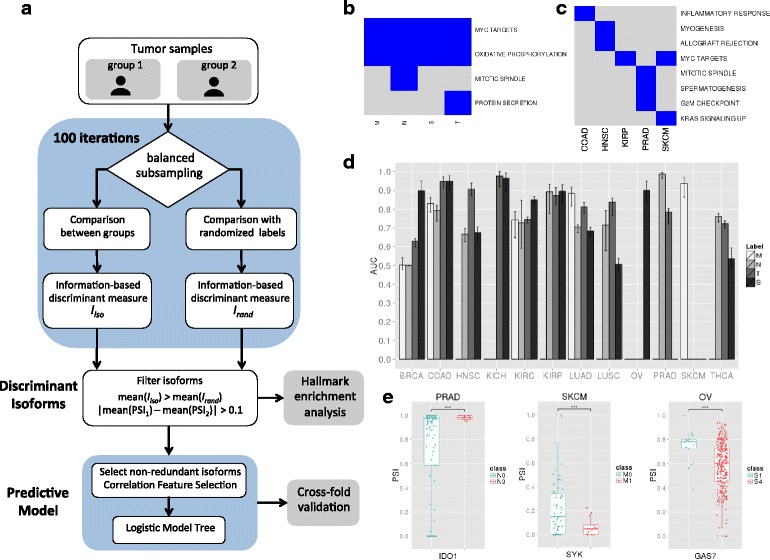
**a** *Workflow* to obtain discriminant transcript isoforms and predictive models. Given two patient groups, we subsampled two equal sized subsets, one from each group (e.g. metastatic and non-metastatic), which were compared using information-based measures, denoted as *I*_*iso*_. At each iteration step, the group labels were randomized to obtain an expected measure, denoted as *I*_*rand*_. After 100 iterations, two distributions were produced for each isoform corresponding to observed (*I*_*iso*_) and expected (*I*_*rand*_) values. Transcript isoforms with a difference of mean PSI values >0.1 in absolute value between the two patient groups and with a positive difference of the means of the observed and expected distributions for all information-based measures used were then considered as discriminant, which were then used to evaluate enriched cancer hallmarks. Discriminant isoforms were further filtered for redundancy with a Correlation Feature Selection strategy to build a predictive model, which was evaluated using cross-fold validation (see “Methods”). **b** Enriched hallmarks in the set of discriminant isoforms for each stage class, metastasis (M), tumor size (T), lymph-node involvement (N), and overall staging (S), using all isoforms selected across all tumor types. **c** Enriched hallmarks for each tumor type using all discriminant isoforms selected across all stage classes in each tumor type independently. **d** Accuracies of the classifiers for each tumor type for the T, N, M, and S annotation, given as the distributions of the areas under the receiving operating characteristic (ROC) curves (AUC). The variation on each *bar* indicates the minimum and maximum AUC values. Some models are absent due to lack of sufficient samples (Table 1). **e** PSI distributions for the transcript isoforms of *IDO1* in PRAD, *SYK* in SKCM, and *GAS7* in OV, for the N-, M-, and S-models, respectively (Wilcoxon test *p* values <0.001)

We considered the standard clinical annotation for tumors based on the tumor size (T), lymph-node involvement (N), metastatic status (M), and combined stage (S) for 4339 patient samples from 12 different tumor types from TCGA (Additional file 1). For each tumor type, we considered the comparison of the transcriptomes between groups of samples in early and late-stage groups according to each stage class independently. That is, for metastasis, we compared non-metastatic samples (M0) against metastatic ones (M1), whereas for the tumor size (T), lymph-node involvement (N), and stage (S) annotations, we compared early and late stages (groups described in Table 1) (see “Methods”). We first calculated the set of transcripts whose relative abundance, measured as percent spliced in (PSI) values, present the best discriminant potential between these groups by using information-based measures with a subsampling strategy to ensure balanced comparisons (Fig. 1a and Additional file 2: Figure S1a). Additionally, we considered only those transcripts that on average change PSI more than 10 % between groups, i.e. |ΔPSI| > 0.1 (see “Methods”). These produced a variable number of transcript isoforms per tumor type and clinical annotation that discriminate between early and late stages or between M0 and M1 (Additional file 3).

**Table 1:** Number of samples analyzed for each tumor type and stage

		T		N		M		S	
Tumor type	Acronym	Early	Late	Early	Late	Early	Late	Early	Late
Breast invasive carcinoma	BRCA	256 (T1)	147 (T3, T4)	455 (N0)	171 (N2, N3)	836 (M0)	15 (M1)	164 (S1)	15 (S4)
Colon adenocarcinoma	COAD	45 (T1, T2)	31 (T4)	149 (N0)	39 (N2)	179 (M0)	33 (M1)	40 (S1)	34 (S4)
Head and neck squamous cell carcinoma	HNSC	35 (T1)	110 (T4)	166 (N0)	166 (N2, N3)			77 (S1, S2)	169 (S4)
Kidney chromophobe	KICH	20 (T1)	19 (T3, T4)					20 (S1)	19 (S3, S4)
Kidney renal clear cell carcinoma	KIRC	245 (T1)	186 (T3, T4)	233 (N0)	16 (N1)	419 (M0)	77 (M1)	240 (S1)	78 (S4)
Kidney renal papillary carcinoma	KIRP	71 (T1)	38 (T3, T4)	23 (N0)	16 (N1, N2)			66 (S1)	38 (S3, S4)
Lung squamous cell carcinoma	LUSC	93 (T1)	59 (T3, T4)	242 (N0)	37 (N2, N3)			195 (S1)	76 (S3, S4)
Lung adenocarcinoma	LUAD	137 (T1)	57 (T3, T4)	281 (N0)	70 (N2, N3)	307 (M0)	22 (M1)	99 (S1)	242 (S3, S4)
Ovarian serous cystadenocarcinoma	OV							18 (S2)	243 (S4)
Prostate adenocarcinoma	PRAD	69 (T2)	93 (T3, T4)	129 (N0)	14 (N1)				
Skin cutaneous melanoma	SKCM					68 (M0)	17 (M1)		
Thyroid carcinoma	THCA	137 (T1)	179 (T3, T4)	220 (N0)	211 (N1)			270 (S1)	48 (S4)

The number of samples used for the comparison early versus late are indicated for each annotation T, N, M, S. Stages I, II, III, and IV are indicated as S1, S2, S3, and S4, respectively. Comparisons were performed between the earliest and latest available stage groups, with some exceptions for which adjacent stages were added to have enough samples for comparison. Empty cells correspond to cases not tested due to lack of sufficient samples or complete lack of annotation in the samples

To characterize the functional involvement of the found discriminant isoforms, we performed an enrichment analysis of cancer hallmarks (see “Methods”) (Additional file 4). Testing discriminant isoforms for each stage class and tumor type independently yielded frequent enrichment of MYC targets, oxidative phosphorylation, mTORC signaling, DNA repair, and Interferon response (Additional file 2: Figure S1b). Notably, aggregating all tumor types for each clinical class, the discriminant transcripts show enrichment in MYC targets and genes involved in oxidative phosphorylation (Fig. 1b). On the other hand, combining discriminant isoforms from different clinical classes in the same tumor type, only five of the 12 tumor types tested show enriched hallmarks (Fig. 1c), which include the enrichment of MYC targets in skin cutaneous melanoma (SKCM) and kidney papillary carcinoma (KIRP). These results indicate that there are frequent transcripts isoform changes in cancer-relevant pathways during tumor progression, many of which may be driven by MYC activity. To test some of our findings, we compared the ΔPSI values of the discriminant transcripts for metastasis in SKCM with the ΔPSI values measured between metastatic (SKMel147) [12] and non-metastatic (Mel505) [13] melanoma cells (see “Methods”). Of the 958 discriminant isoforms in SKCM, 817 had expression in the cell lines. From these, 504 (61.7 %) show a change in PSI in the same direction and 253 of them have |ΔPSI| > 0.1 in both comparisons (Additional file 2: Figure S1c).

To build signatures of tumor stage based on transcript isoforms, we applied a multivariate feature selection method on the discriminant isoforms selected before to obtain a non-redundant subset of predictive transcripts, which we used to build logistic model trees (LMT) for each tumor type and stage class (Fig. 1a) (models given in Additional file 5). Each one of these models represents a transcript signature for each stage class and each tumor type. Using cross-validation on the annotated TCGA samples, the mean accuracy of the models in terms of the area under the ROC curve (AUC) is 0.783 (Fig. 1d), with similar average precision-recall values (Additional file 2: Figure S1d). T-models show the best accuracies (mean AUC = 0.824), with the models for KIRP, kidney chromophobe (KICH), colon adenocarcinoma (COAD), and neck squamous cell carcinoma (HNSC) being the most accurate (mean AUC >0.87).

The KIRP T-model includes an isoform for *PAX6*. Increased inclusion of exon 5 of this gene has been related to neuronal differentiation [14], which we see associated with the late T stage (Additional file 2: Figure S2a). The best N-models correspond to KIRP and prostate adenocarcinoma (PRAD) (mean AUC >0.89). The KIRP N-model includes an isoform in the MAP kinase *MKNK1* (Additional file 2: Figure S2a), suggesting a similar involvement in cancer as *MKNK2* [15]. The PRAD N-model (mean AUC = 0.986) includes an isoform of *IDO1* (Fig. 1e), a gene related to anti-tumor defense [16]. The best M-model corresponds to SKCM (mean AUC = 0.93) and includes an isoform change in the transmembrane gene *TM6SF1* (Additional file 2: Figure S2a) and the tyrosine kinase *SYK* (Fig. 1e). In metastatic melanoma samples, *SYK* shows an increase in the abundance of the long form and a decrease of the short form, as previously observed in breast tumors [17]. Finally, the best S-models correspond to COAD, breast invasive carcinoma (BRCA), KICH, and ovarian serous cystadenocarcinoma (OV) (AUC >0.9). Interestingly, OV S-model includes an isoform in the cancer driver *GAS7* (Fig. 1e). In general, we found no overlap between the different stage models. A notable exception is an isoform of *NSUN7* that appears in all models for kidney renal clear cell carcinoma (KIRC) with high PSI values at late-stage and an isoform of *SKA3* that appears in the N-, T-, and S-models for KIRP, with low PSI values at late stages. The low general overlap is consistent with pathological transformations being associated with multiple molecular alterations.

### Transcript isoform changes are predictive of survival in patients with unknown stage

**Fig. 2:**
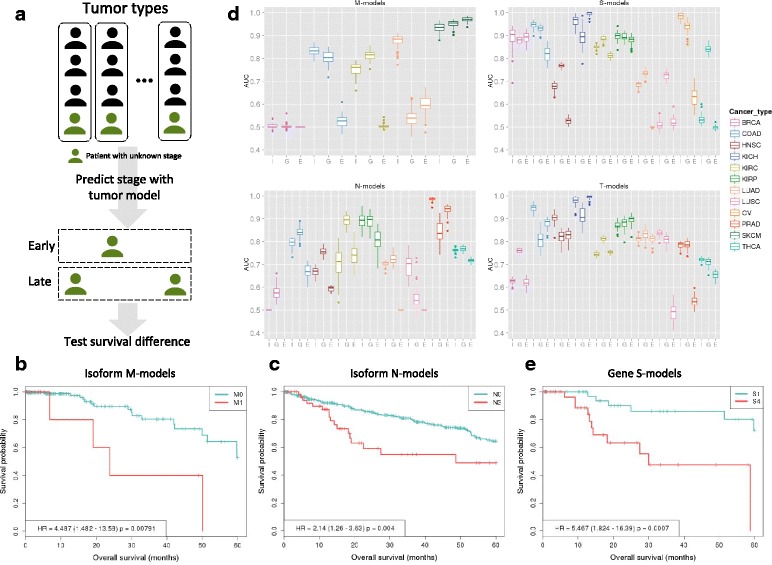
**a** *Illustration* of the blind test on unlabeled patients. Patients without annotated stage were predicted using the model of the corresponding tumor type, for each of the stage classes independently. Patients predicted as early or late were collected into two separate groups and tested for differences in survival. This test was performed for each stage class independently and only using tumor types that showed an association between stage and survival in the labeled patients (Table 2). **b**, **c** *Survival (Kaplan–Meyer) plots* associated with the test for M-models and N-models, respectively. They indicate the survival percentage (*y-axis*) versus survival in months (*x-axis*) based on the predicted stage on the unannotated samples using the classifier for each corresponding tumor type. The *p* value in each *plot* corresponds to the Cox regression between the two groups and HR indicates the hazard ratio. **d** Accuracies of the transcript isoform models (I) compared to the gene (G) and event (E) models. Accuracies are given as *boxplots* for the distribution of AUC values (*y-axis*) from a tenfold cross-validation for each tumor type (*x-axis*) for the M-, S-, N-, and T-models. Tumors for which stage data were missing are not shown (Table 1). **e** *Survival (Kaplan–Meyer) plot* of the early and late-stage predictions performed with the gene-based S-models on unannotated samples. The *p* value corresponds to the Cox regression between the two groups. *HR* hazard ratio

We hypothesized that if the derived transcript signatures provide clinically relevant information, we should find worse clinical outcomes for patients predicted to be at a late stage. We thus performed a blind test on those samples that lacked stage annotation, and therefore were not used for building the models, to predict the tumor stage using the model for the corresponding tumor type (Fig. 2a and Additional file 1). Additionally, we only performed the blind test in those tumor types for which late clinical stage was significantly associated with a worse prognosis in the labeled samples (Table 2). There were 40 samples from COAD, 116 from lung adenocarcinoma (LUAD), and 80 from BRCA that lacked M annotation. After prediction with the M-model from each tumor type, we obtained a total of 226 patients predicted as M0 and 10 patients predicted as M1. Aggregating patients according to the predicted metastatic class yielded a significant difference in survival between the two groups (*p* value = 0.0079) (Fig. 2c). Regarding lymph node invasion, there was one sample from COAD, 10 from LUAD, 82 from KIRP, 247 from KIRC, and 74 from HNSC without N annotation. After predicting with the N-models from the corresponding tumor types, 356 and 58 patients were predicted as early and late N, respectively. Survival analysis with the aggregated patients yielded a significant difference between the two predicted groups (*p* value = 0.013) (Fig. 2d). Finally, for the S stage, we predicted on a set of 91 samples without S annotation (eight from COAD, 18 from BRCA, 47 from HNSC, 11 from KIRP, four from lung squamous cell carcinoma (LUSC), two from thyroid carcinoma (THCA), and one from LUAD). This resulted in 47 and 44 samples predicted as early and late, respectively, which showed no difference in survival (*p* value = 0.479). These results represent an independent validation of our transcript signatures and provide evidence that the relative abundances of transcripts may hold some predictive value for tumor staging and prognosis.

**Table 2:** Survival analysis between early and late-stage patient groups

Tumor type	T	N	M	S
BRCA	*p* = 0.375	*p* = 0.00012	*p* = 0.008	*p* = 0.0007
COAD	*p* = 0.0011	*p* = 0.011	*p* = 1.48e-05	*p* = 0.012
HNSC	*p* = 0.051	*p* = 0.0137		*p* = 2.49e-07
KICH	*p* = 0.00896			*p* = 0.00896
KIRC	*p* = 2e-15	*p* = 0.0125	*p* = 0	*p* = 0
KIRP	*p* = 0.0043	*p* = 0.005		*p* = 8.86e-007
LUSC	*p* = 0.029	*p* = 0.071		*p* = 0.025
LUAD	*p* = 7.02e-09	*p* = 3.26e-06	*p* = 0.165	*p* = 7.02e-09
OV				*p* = 0.0537
PRAD	*p* = 0.456	*p* = 1		
SKCM			*p* = 0.418	
THCA	*p* = 0.324	*p* = 0.597		*p* = 2.49e-07

*p* values from the survival test comparing the patient subsets from Table 1. The *p* values were obtained using a Cox proportional hazards regression model. Empty cells correspond to cases not tested due to lack of sufficient samples (see Table 1)

### No relation of isoform signatures with stromal and immune cell content

To assess whether the purity of the samples could be a potential confounding factor of the derived signatures, we tested the correlation between the transcript PSI values of our models against signatures of stromal and immune cell content [18] (see “Methods”). Overall, all signatures showed low correlation with stromal content (mean Pearson |R| <0.4), and all except the N-model in BRCA (Pearson R = 0.433) had mean |R| <0.4 with immune cell content (Additional file 6). From the 547 transcript isoforms tested, 95 % show a correlation |R| <0.4 (Pearson) for both stromal and immune scores. Among the few cases with |R| >0.5 there is an isoform of *ENAH* (Additional file 2: Figure S2d), which is present in the T-models in KIRP and COAD and that was previously linked to an invasive phenotype [19]. Recent analyses have shown that clinical stage does not correlate with tumor purity in the TCGA samples [20]. Our analysis further supports those results and indicates that isoform-based signatures of stage do not reflect stromal or immune cell content.

### No universal transcript isoform signature for tumor staging

Our results prompt the question of whether there might be a universal signature of stage and survival based on transcript isoform changes. To test this, we grouped all annotated samples from the different tumor types according to the stage class and applied the same analyses as before. We could only build M- and S-models due to the lack of common isoforms with discriminant power for the other classes (Additional file 7). The average AUC values for M- and S-models were lower than before, with mean AUC of 0.5 and 0.685, respectively. Aggregating samples from BRCA, COAD, and LUAD, we observed a slight increase in accuracy (mean AUC = 0.702). Similarly, analyzing KIRC, KIRP, and KICH samples together, the S-model achieves mean AUC = 0.809. In this case, approximately half of the isoforms were present in the previous models. Finally, analyzing the squamous tumors together (HNSC and LUSC), we derived N- and S-models with mean AUC = 0.72. For other combinations, we could not find accuracies greater than AUC = 0.5. This indicates that despite some overlapping features across tumor types, there is no common signature for all the tested tumor types.

### Transcript signatures provide better predictions than event-based signatures in retrospective datasets

We tested whether local alternative splicing events, as opposed to transcript isoform changes, could also determine stage. We applied our analysis pipeline using PSI values for all events in the same tumor samples used before. For most of the stage classes we observed similar or smaller accuracy values for events compared to transcript models (average AUC 0.617 versus 0.778, respectively) (Fig. 2d and Additional file 8). Only 23.5 % of the isoforms in models overlap with at least one alternative splicing event from the event-based models: 16.51 % overlap with alternative 5′/3′ splice-sites, mutually exclusive exons, retained introns, or cassette exon events, and 6.54 % overlap with alternative first or last exon events. Moreover, 82.39 % of isoforms in models overlap with at least one of the pre-calculated alternative splicing events. This indicates that a considerable number of changes in exon-intron structures described by the isoform models that are predictive of tumor stage cannot be captured in terms of simple alternative splicing events.

### Transcript signatures provide relevant information about tumor metastasis and lymph node invasion independently of gene expression

Previously proposed molecular classifiers of stage were based on gene expression [7, 8]. We thus tested the relation of our transcript signatures with gene expression. We observed that the proportion of genes with differential expression (DE) vary markedly between transcript signatures (Additional file 5 and “Methods”). For M-models, nine (18 %) genes in the SKCM and four (18 %) genes in KIRC showed DE. For N-models, we only found three (14 %) in PRAD and 13 (68 %) in THCA. In contrast, T-models presented frequent changes across the different tumor types, with 17 (46 %) in KIRP, seven (27 %) in KIRC, six (33 %) in LUAD, four (50 %) in THCA, and one in HNSC (5 %). Similarly, S-models also showed frequent DE: 16 (52 %) in KIRC, 12 (46 %) in KIRP, one (25 %) in LUAD, and one (7 %) in BRCA.

Next, we compared the discriminative power of transcript and gene expression signatures. We thus applied our pipeline to gene expression values to derive gene-based signatures of stage (see “Methods”). The overall accuracy for gene-based signatures was similar to isoform-based models (average AUC values 0.783 and 0.781 for isoforms and genes, respectively) (Fig. 2d and Additional file 9). Interestingly, isoforms had better mean accuracies for the M-model in LUAD (0.883 versus 0.535) (Fig. 2d, upper left panel) and for the N-model in PRAD (0.986 versus 0.839) (Fig. 2d, lower left panel), compared to gene models. In contrast, the gene-based S-model for THCA showed higher accuracy (0.529 versus 0.836) (Fig. 2d, upper right panel). Gene and isoform based models generally involved different genes with only few exceptions, including *CD72* in SKCM M-models, *PTGS2* and *VIPR1* in the THCA T-models, *SLC14A1* in COAD S-models, and *DNASE1L3* KICH S-models. Interestingly, gene-based S-models were predictive of survival for samples lacking stage S annotation (*p* value = 0.0024) (Fig. 2e), whereas no significant difference in survival was found with the gene-based M- and N-models (*p* values = 0.983 and 0.161, respectively).

The results described above suggest that genes and transcripts provide independent information and may yield better signatures when combined together. We thus built mixed models of gene expression and transcript relative abundance. We started with all gene and transcript discriminant features and selected a non-redundant set of features to build logistic-model trees. The accuracy of these mixed models was on average better (mean AUC = 0.831) than using only transcripts or genes (Additional file 2: Figure S3a). Notably, the transcript signatures performed better than the mixed signatures for the LUAD M-model (AUC = 0.883 versus 0.814) or the PRAD N-model (AUC = 0.986 versus 0.938). In contrast, the mixed model performed better in the COAD M-model (AUC = 0.831 versus 0.864) and the HNSC S-model (AUC = 0.676 versus 0.778). Additionally, mixed models are able to predict survival differences between early and late stages for the N and S stage classes (*p* value = 0.041 and 0.033, respectively) (Additional file 3: Figure S3b and S3c).

Finally, we compared our transcript signatures with an expression signature of 44 genes built to differentiate metastatic and late-stage samples in colon cancer [21] (see “Methods”). The mean AUC values obtained for the metastatic annotation (M) and the overall stage (S) were 0.612 and 0.649, respectively, for the gene expression signature and 0.82 and 0.94 for our transcript signatures. Notably, none of the genes involved in our transcript models for COAD presented DE. Our analyses indicate that changes in the relative abundance of transcripts hold relevant information about tumor transformation independently of gene expression changes.

### Transcript relative abundances as prognostic markers in ER-negative breast tumors

**Fig. 3:**
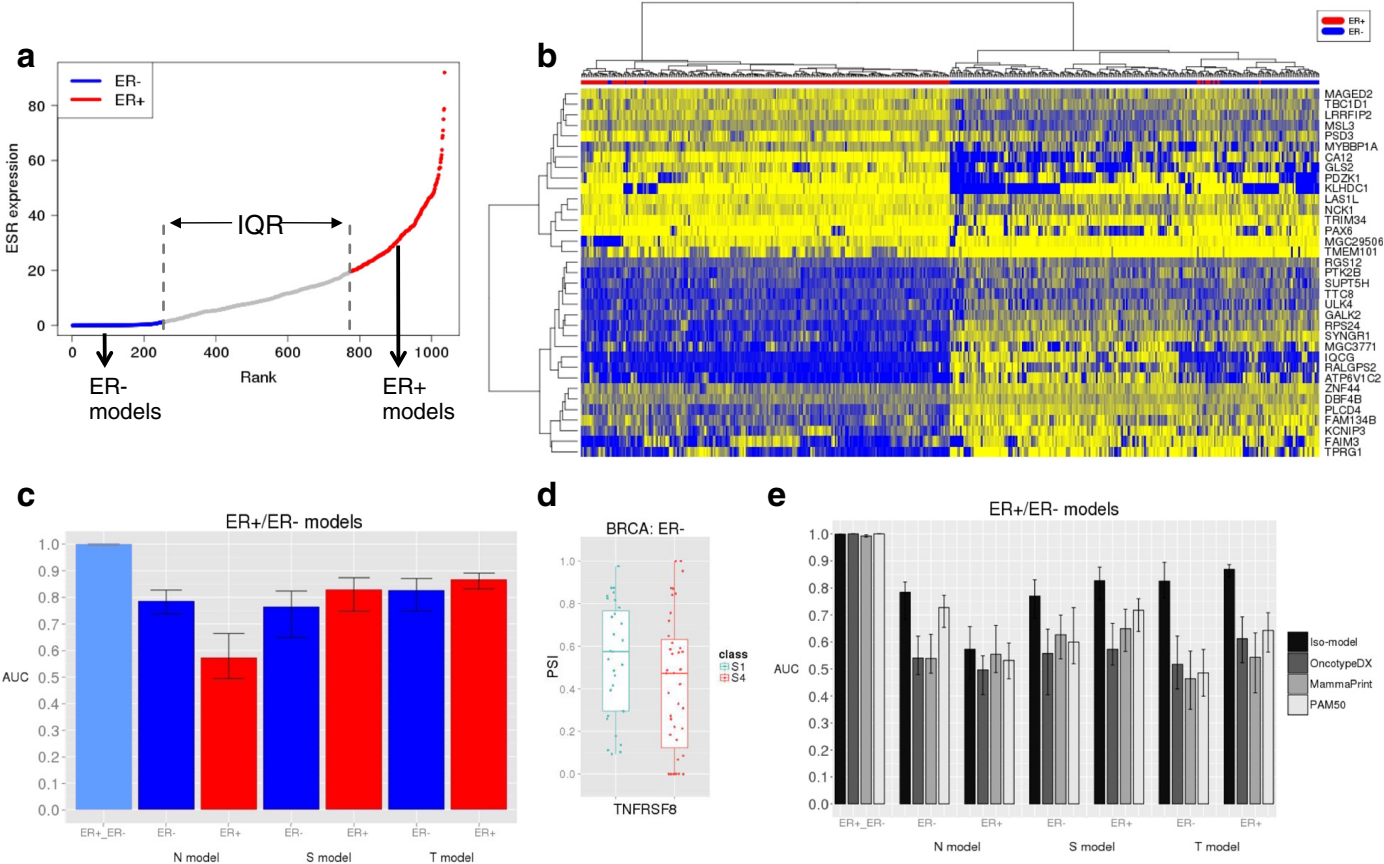
**a** Ranking (*x-axis*) of breast tumor (BRCA) samples according to *ESR1* expression (gene TPM) (*y-axis*). ER+ and ER– subsets were defined as the top and bottom 25 % of the ranking, respectively, leaving out samples in the interquartile range (IQR). **b** *Heat map* of PSI values, from 0 (*blue*) to 1 (*yellow*), for the top 35 isoforms that separate ER+ and ER– subsets. Isoforms are labeled by gene name (*y-axis*). Samples are clustered according to the PSI values using Euclidean distance and Ward’s method. **c** Accuracies in terms of AUC values (*y-axis*) from a tenfold cross-validation for the transcript isoform signatures for the comparison of ER+ and ER– samples, and for the comparison of early and late N, S, and T stages within ER+ or ER– subsets. The variation on each *bar* indicates the minimum and maximum AUC values. **d** PSI distribution of the isoform in *TNFRS8* that changes between early and late S stage in ER– samples (Wilcoxon test *p* value = 0.1046). **e** Accuracies in terms of AUC values (*y-axis*) from a tenfold cross-validation for the transcript isoform signatures (Iso-model) and the gene expression signatures OncotypeDX, MammaPrint, and PAM50, indicated in *grayscale*. Each signature was tested to predict the separation of ER+ and ER– breast tumor samples or the separation between early and late (N, S, and T) stage in ER+ or ER– separately. The variation on each bar indicates the minimum and maximum AUC values

Molecular subtypes in cancer have implications for prognosis and therapy that go beyond the staging system [6, 22, 23]. In breast cancer, tumors that are negative for the estrogen receptor (ER) have a generally worse prognosis and gene expression signatures are generally less accurate for ER-negative than for ER-positive tumors [3, 7]. To test whether transcript-based signatures could be relevant for ER-negative tumors, we separated the samples according to the expression ranking of the ER gene (*ESR1*) into the top (ER+) and bottom (ER–) 25 % (237 samples each) (Fig. 3a). Interestingly, applying our pipeline we identified 2591 discriminant transcript isoforms between the ER+ and ER– subgroups (Fig. 3b and Additional file 9). These transcriptome changes were validated using RNA-seq data from the knockdown of *ESR1* and control in MCF7 cells [24] (Additional file 2: Figure S4a and “Methods”). We derived a predictive model with 81 discriminant transcripts that separated ER+ and ER– samples with an average AUC of 0.999 (Fig. 3c). Among the largest PSI changes, we found an isoform of the MAP kinase *MAP3K7*, whose long isoform was linked before to apoptosis [25], which we found to be less abundant in ER– samples (Additional file 2: Figure S4b). Notably, 47 (58 %) of the genes with transcripts in this model show DE, suggesting a link between ER expression and the differential use of transcript isoforms.

The observed transcriptome differences between ER+ and ER– subtypes warrant a separation of these two sets to build transcript signatures of stage. Accordingly, we considered early and late-stage patients in each ER group separately (Table 3). Since ER– samples show significant differences in survival between early and late stages for N (*p* value = 0.005) and S (*p* value = 0.041) annotations (Additional file 2: Figure S4c and S4d), we expect that a signature for stage may be relevant for prognosis. In contrast, ER+ samples do not show any significant differences in survival. Using our feature selection pipeline, we obtained 456 and 249 isoforms that best discriminate between early and late stages in the ER– and ER+ subsets, respectively (Additional file 9). The isoforms for ER– show enrichment in various cancer hallmarks, including DNA repair, apoptosis, and epithelial-mesenchymal transition (Additional file 2: Figure S4e). In contrast, there were no enriched hallmarks associated with the isoforms in the ER+ subset. Building stage signatures as before for ER+ and ER– independently (Additional file 9), we obtained average accuracies of AUC = 0.794 (ER–) and AUC = 0.756 (ER+) (Fig. 3c), with similar values for the precision-recall (Additional file 2: Figure S4f). Notably, none of the derived signatures showed DE at the gene level. Additionally, the ER– S-model includes *TNFRS8* (Fig. 3d), a member of the tumor necrosis factor receptor superfamily. Another member of this family, *TNFRSF17*, was related before to prognosis in the ER– samples [3]. Unlike for the previous models, there were not enough unlabeled samples to perform a blind test. Taken together, these results show that transcript variants can be informative for stage and prognosis in ER-negative tumors.

**Table 3:** ER-negative (ER–) and ER-positive (ER+) breast tumor subgroups

	T		N		S	
BRCA subtype	Early	Late	Early	Late	Early	Late
ER–	72 (T1)	48 (T3, T4)	122 (N0)	37 (N2, N3)	48 (S1)	55 (S3, S4)
ER+	54 (T1)	29 (T3, T4)	130 (N0)	36 (N2, N3)	31 (S1)	43 (S3, S4)

The number of samples used for the early vs. late comparison is indicated for each annotation T, N, and S. Stages I, II, III, and IV are indicated as S1, S2, S3, and S4. In some cases, more than one clinical stage is included in a patient group to have sufficient samples. Due to the insufficient number of annotated samples, it was not possible to build M-models

We further compared our transcript signatures with known gene expression signatures for breast tumors: OncotypeDX [26]; MammaPrint [27]; and PAM50 [28] (see “Methods”). Although these signatures were not originally designed to identify tumor stage, they bear predictive value for this purpose [7]. Their accuracies to separate the ER+ and ER– subgroups were very similar to our transcript signatures (Fig. 3e). This is expected for PAM50 and OncotypeDX, as they include *ESR1*. We then tested how well the gene signatures differentiate stage within each subset, ER+ or ER–, independently. In general, PAM50 performed better than the two other signatures, except for S in ER– and for N in ER+, where MammaPrint performs better, and for T in ER–, where OncotypeDX performs better (Fig. 3e). Notably, in all cases the transcript signature had better accuracies. We conclude that transcript isoform models can provide relevant information to determine stage and hence complement current clinical signatures.

### Transcript relative abundances characterize an invasive phenotype and survival in melanoma

**Fig. 4:**
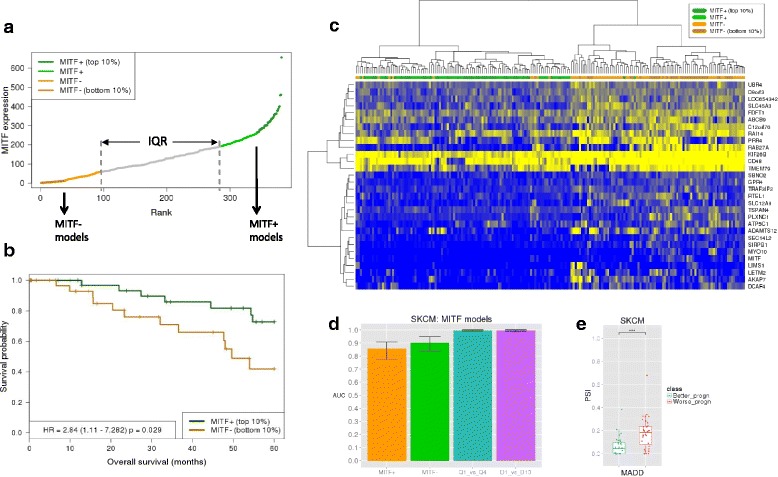
**a** Ranking (*x-axis*) of melanoma (SKCM) samples according to *MITF* expression (gene TPM) (*y-axis*). We indicate the top and bottom 10 % and 25 % of the samples used for analyses. **b** *Survival (Kaplan–Meyer) plot* for the top and bottom 10 % of the samples according to the ranking of *MITF* expression. The *p* value corresponds to the Cox regression between the two groups. *HR* hazard ratio. **c** *Heat map* of PSI values, from 0 (*blue*) to 1 (*yellow*), for the top 30 discriminant isoforms according to |ΔPSI| value between the MITF+ and MITF– subgroups. Isoforms are labeled by gene name (*y-axis*). Samples are clustered according to the PSI values using Euclidean distance and Ward’s method. **d** Accuracy given in terms of the distribution of AUC values (*y-axis*) from a tenfold cross-validation for (from left to right in the *x-axis*) the survival model for MITF+, MITF– as well as for the separation between MITF+ and MITF– subgroups using 25 % (Q1 vs. Q4) or 10 % (D1 vs. D10) of the top and bottom samples in the ranking of *MITF* expression. The *bars* show the minimum, mean, and maximum AUC values. **e** Distribution of PSI values for the isoform in *MADD* that is predictive of prognosis in the MITF– subgroup (Wilcoxon test *p* value = 7.781e-05)

Clinical outcome of SKCM remains poor due to its high degree of heterogeneity [29]. The microphthalmia-associated transcription factor (*MITF*) presents highly dynamic expression patterns in connection to proliferation and invasion in melanoma, with relevance for prognosis and therapy [30, 31]. Overexpression and downregulation of *MITF* have been connected to proliferative and invasive phenotypes, respectively [32]. We thus tested whether there are specific transcript signatures linked to these phenotypes that could be linked to survival. We pooled the top and bottom 25 % of melanoma samples according to *MITF* expression into the MITF+ and MITF– sets, respectively (96 samples per set) (Fig. 4a). Although these subsets do not show a significant difference in survival, samples in the top and bottom 10 % of *MITF* expression (36 samples per set) show a significant difference, with *MITF* overexpressed samples showing worse prognosis (*p* = 0.029) (Fig. 4b). Our feature selection strategy (Fig. 1a) yielded 2387 discriminant isoforms between MITF+ and MITF– (Fig. 4c and Additional file 9). We validated these isoforms by comparing their ΔPSI values with those obtained from the knockdown of *MITF* in melanoma cells compared to controls [13] (Additional file 2: Figure S5a and “Methods”). The found discriminant isoforms are enriched for multiple cancer hallmarks, including EMT and the mTOR pathway (Additional file 2: Figure S5b). To further characterize their differences, we built a model to separate MITF+ and MITF– samples with 72 isoforms, which showed a mean AUC of 0.996 (see “Methods”). This model included a transcript isoform for the cancer driver *TPM1*, which is highly included in MITF+ and was linked before to tumor growth [33] (Additional file 2: Figure S5c), as well as for *RAB27A*, a component of the melanosome that is transcriptionally regulated by *MITF* [34] and that is lowly included in MITF+ samples (Additional file 2: Figure S5d). From this signature, 37 (66 %) of the genes involved showed DE between MITF+ and MITF– subgroups, pointing to a link between *MITF* expression and differential usage of transcript isoforms in multiple genes.

To test whether the melanoma phenotypes are associated with different transcript transformations during tumor progression, we studied the MITF+ and MITF– sets independently to derive signatures of survival. We selected samples in the top and bottom 40 % according to days of survival (36 samples per group) and used our pipeline to calculate the isoforms that best separate these groups within each phenotype. The discriminant isoforms in the invasive phenotype (MITF–) were enriched for multiple cancer hallmarks, whereas the proliferative phenotype (MITF+) presented enrichment only for activation of *KRAS* signaling, which does not appear in the invasive phenotype (Additional file 2: Figure S5b). We then built models of survival for each subset independently using LMTs (Additional file 9). Cross-validation yielded for MITF+ (34 isoforms) and MITF– (46 isoforms) accuracies of AUC = 0.854 and 0.896, respectively (Fig. 4d and Additional file 2: Figure S5e). Notably, the MITF– model includes a transcript isoform for the MAP Kinase-Activating Death Domain gene *MADD* (Fig. 4e), which does not change expression at the gene level. *MADD* is a cancer driver and it was shown before that expression of isoforms that skip exon 16 has anti-apoptotic effects [35]. Interestingly, the PSI of the *MADD* isoform that skips exon 16 is higher in the group with worse prognosis, suggesting that the anti-apoptotic function of *MADD* is related to worse prognosis in invasive melanoma. Taken together, our results provide evidence of distinct transcript abundance patterns linked to melanoma phenotypes and survival.

## Discussion

We described the first systematic analysis of the potential of transcript relative abundances to determine stage and clinical outcome in multiple solid tumors. We derived novel molecular signatures for 12 different tumor types that can separate tumors according to clinical stage or metastatic status in retrospective datasets. Importantly, a blind test on patients with unknown stage or metastatic status can separate patients according to survival. Moreover, transcript isoforms provide better accuracies than local alternative splicing events and can describe more complex changes in exon-intron structures. Although a multi-cancer signature of clinical outcome based on gene expression has been proposed [36], our results argue against a generic transcript-based signature for all tumors types. Rather, transcript isoform changes appear linked to tumor-type specific processes, with several of them related to MYC activity, in concordance with recent findings [37].

We observed a widespread association between transcript isoform changes and expression changes in T- and S-models across tumor types and around 60 % of the genes with DE in all models correspond to KIRC and KIRP, indicating that transcript and gene changes are tightly coupled during progression of these tumors. In contrast, this association is low or absent for most tumor types for M- and N-models. The blind test showing that patients with predicted metastasis or late N-stage have a worse prognosis was for tumor types for which none of these models show gene expression differences. The value of the transcript signatures is further highlighted when compared to known and newly derived gene expression signatures or with mixed models combining gene expression and transcript abundances. These results indicate that transcripts signatures provide information independent from gene expression to describe tumor progression and especially in metastasis and lymph node invasion.

We also extracted signatures for specific tumor subtypes in breast cancer and melanoma. We reported many significant transcript isoform changes between breast tumors according to ER expression and between melanoma samples according to *MITF* expression. Additionally, we observed a widespread association between transcript isoform and expression changes in relation to ER expression and according to *MITF* expression. An interesting possibility is that the activity of these two transcription factor genes could trigger expression and transcript isoform changes in the same genes in these tumors, pointing to new mechanisms of gene regulation worth investigating further. We further derived transcript signatures of stage independently in each sample subset that involved different genes, thereby highlighting the relevance of determining the transcriptome repertoire in tumor samples to derive accurate molecular signatures of tumor progression.

We observed partial reproducibility of the discriminant isoforms in experiments using cell lines. Transcriptional differences between cell lines and tumor tissues are thought to stem from the loss of the stromal and immune components by cells in culture [38]. Our analyses discard an association between the transcript signatures and the composition of stromal and immune cells in the tissue samples. It could be possible that part of the signatures reflects the interaction of tumor cells with their environment in tissues samples, which would be undetectable in cell lines. Our results support the notion that phenotypic states of tumor cells, like invasiveness, may be reflected on the relative abundance of transcript isoforms and may be partly triggered by external cues, such as inflammation or metabolic stress [39]. On the other hand, the observed commonalities between tumor cells and tissues suggest that some of these alterations could be investigated further using cell lines.

The clinical validity of our findings remains to be tested. Our cross-fold validation in retrospective datasets shows good accuracies in general for transcript signatures and, in particular, isoform-based M- and N-models are generally more accurate compared to gene expression models on the same datasets. Additionally, we have shown that predicted metastatic and late lymph-node involvement in non-annotated patients is associated with worse prognosis. These results thus suggest that isoform models may be potentially useful to indicate metastasis or lymph node invasion. To conclusively establish the validity of these signatures and their value to improve current methods of stage determination, it would be necessary to perform more validations on independent cohorts and to verify that they provide stage information before it is visible by other means in prospective studies. However, further studies are currently hampered by the scarcity of large enough RNA-seq datasets with clinical annotation comparable to TCGA [40].

## Conclusions

Our analyses reveal that transcript isoforms hold useful information to build signatures of stage and clinical outcome independently of gene expression. Transcript isoform signatures appear especially relevant to determine lymph node invasion and metastasis. We anticipate that these and similar models may become relevant to understand the progression of tumors beyond DNA and gene expression alterations, thereby complementing current molecular approaches in precision cancer medicine.

## Methods

### Datasets

Processed RNA-seq data from TCGA (https://gdc.nci.nih.gov/) was compiled for 12 different tumor types: BRCA, COAD, HNSC, KICH, KIRC, KIRP, LUAD, LUSC, PRAD, SKCM, THCA, and OV. The abundance of every transcript per sample was calculated in transcripts per million (TPM) from the transcript-estimated read counts and the isoform lengths. Genes were defined to be a set of transcripts that overlap in the same genomic locus and strand and share at least one splice-site (Additional file 1). A gene TPM was defined as the sum of TPMs for all transcripts in the gene. The relative abundance of each isoform (PSI), was calculated by normalizing the isoform TPM to the gene TPM. Only genes with a minimum TPM of 0.1 were considered. Additionally, we used RNA-seq data from the knockdown of *ESR1* and controls in MCF7 cells (GSE53533) [24], from metastatic melanoma cells (SKMel147) and melanocytes (GSE68221) [12], and from the knockdown of *MITF* and controls using non-metastatic melanoma cells (Mel505) (GSE61967) [13]. For each sample, transcript abundances were calculated with Sailfish [41]. Relative abundances (PSI) of transcripts were calculated as above and the ΔPSI values between conditions were calculated as the difference between conditions of the mean values from the replicates. Alternative splicing events and their PSI values were obtained from [42].

### Clinical data

Clinical stage and survival information for patients was obtained from TGCA. We used the available annotation for the TNM staging system (www.cancerstaging.org/), where T followed by a number (1–4) describes the size of the tumor; N followed by a number (1–3) describes spread to lymph nodes according to number and distance; and M followed by 1 or 0 indicates whether the tumor has metastasized or not, respectively. We also considered the numbered stage annotation (S), which goes from 0 to 4, with each number corresponding approximately to a combination of the TNM numbers. When any of the stages were subdivided, only the label of the common class was included (e.g. T1a, T1b, and T1c were considered as T1). Only patients with defined stage were used to build the predictive models.

### Selection of relevant features

Only isoforms and events with a difference in mean relative abundance (PSI) of at least 0.1 in absolute value between the compared patient subgroups were considered to calculate discriminant isoforms. To obtain discriminant genes, those with log-fold change of the mean gene TPM values between the two groups greater than 2 were considered. Next, a subsampling approach was used to compare two patient groups through 100 iterations, by extracting the same number of samples from each group randomly from the input dataset, using a minimum of 10 samples per group. For pooled tumor types, the same number of samples per tumor type was selected at each iteration step. At each iteration step, three different univariate discriminant measures were applied (see below), and a permutation of the group labels was performed and the univariate measures re-calculated. After 100 iterations, and for each univariate measure, two distributions of 100 points each are produced for each transcript, corresponding to the observed and expected values. Transcripts with a positive difference of the means of the two distributions for all three measures were considered discriminant and were kept for further analysis.

We applied the following information-based measures in the subsampling: information gain (*IG*), gain ratio (*GR*), and symmetrical uncertainty (*SU*). *IG* is defined as the mutual information between the group labels of the training set *S* and the values of a feature (or attribute) A, e.g. an isoform: *IG(S,A) = MI(S,A) = H(S) - H(S|A)*, where *H(S)* is Shannon’s entropy according to the two sample classes and *H(S|A)* is the conditional entropy of *S* with respect to the attribute *A. GR* is the mutual information of the group labels and the attribute, normalized by the entropy contribution from the proportions of the samples according to the partitioning by the attribute: *GR(S,A) = MI(S,A)/H(A)*. Finally, *SU* provides a symmetric measurement of feature correlation with the labels and it compensates possible biases from the other two measures: *SU(S,A) = 2 · MI(S,A)/(H(S) + H(A))* [43]. The group labels are the clinical stages (early, late), survival groups (low, high), or phenotype group (invasive, proliferative); and the attribute values are the PSI values for transcript isoforms or alternative splicing events or the gene TPM values for gene expression analyses. The continuous PSI or TPM values were discretized as previously described [44].

### Cancer hallmarks and drivers

Enrichment analysis of the 50 cancer hallmarks from the Molecular Signatures Database v4.0 [45] was performed with the discriminant isoforms. For each hallmark, Fisher’s exact test was performed with the genes with selected isoforms using as controls genes expressed (TPM >0.1) and with multiple transcripts. A Benjamini–Hochberg correction was applied and only cases with a false discovery rate <0.05 were kept. Known and predicted cancer drivers were obtained as described in [42].

### Transcript signatures

Transcript isoforms that showed a positive difference between the means of the 100 observed and the 100 randomized values for all three univariate measures (*IG*, *GR*, *SU*) were analyzed with a Correlation Feature Selection (*CFS*) [43]. This selects transcripts with similar discriminating power but lower redundancy among them [43], thereby mitigating the problem of overfitting. This was repeated for each comparison between clinical stages, survival groups, or tumor subtypes. Using the selected transcript isoforms, an LMT was built with Rweka [46]. LMTs are classification trees with logistic regression functions at the leaves. The accuracy of the classifiers was evaluated using the AUC. Additionally, we considered the area under the precision-recall curve (PRC). AUC and PRC take values between 0 (worst prediction) and 1 (best prediction). These values were estimated for each classifier through a tenfold cross-validation, repeated 100 times. The same approach was used for gene, event, and mixed models. To apply known gene expression signatures to our sample groups we used robust Z-scores per gene and per sample as described before [42]. These values were then used for the genes in various signatures [21, 26–28]. As before, accuracies were estimated using a tenfold cross-validation to calculate AUC and PRC values.

### Blind tests

For samples without stage annotation, which were not used to build the models, we predicted the missing stage (early/late) or metastatic state, using the corresponding model for the same tumor type. These newly predicted samples were then aggregated per clinical class according to early and late, or metastatic and non-metastatic, to test the survival differences between groups. The blind test was performed using only those tumor types that already showed significant differences in the survival between early and late stages for the annotated samples (Table 2). This analysis was not performed for T-models, as all samples had a T annotation.

### Differential expression analysis

We performed DE analysis for all genes between the different groups considered in this analysis, using the same method as described previously [42]. Genes were considered differentially expressed if the absolute value of the log2-fold change was greater than 0.5 and corrected *p* value < 0.05. Results can be found in Additional files 5 and 9.

### Survival analysis

Survival curves were calculated with the Kaplan–Meier method and compared between patient subsets using a Cox proportional hazards regression model [47]. Survival was measured as date of death minus collection date for deceased patients and as last contact date minus collection date for the other patients.

### Stromal and immune cell content analysis

To estimate a stromal and immune signature for a set of samples from a tumor type, we collected a list of stromal and immune signature genes based on [18]. We transformed the RNA-Seq by Expectation-Maximization (RSEM) read counts of these two gene lists into a gene set score using gene set variation analysis (GSVA) [48] for each sample. Using the resulting scores per sample, we then calculated the Pearson correlations of the stromal and immune GSVA scores with the transcript isoform PSIs using all tumor samples, including intermediate stages.

## Abbreviations

AUC, area under the ROC curve; CFS, correlation feature selection; ER, estrogen receptor; GR, gain ration; IG, information gain; LMT, logistic model tree; PRC, area under the precision-recall curve; PSI, percent/proportion spliced in; ROC, receiver operating characteristic; SU, symmetrical uncertainty

## Additional files

Additional file 1: Patient data, blind predictions, and gene-isoform annotations. (XLS 4554 kb) Additional file 2: This additional file contains the supplementary figures cited in the paper. (PDF 1828 kb) Additional file 3: Discriminant transcript isoforms between stages and patient groups. (XLS 4562 kb) Additional file 4: Enriched cancer hallmarks. (XLS 66 kb) Additional file 5: Transcript signatures of tumor stage (XLS 189 kb) Additional file 6: Correlation of predictive isoforms with stromal and immune scores. (XLS 173 kb) Additional file 7: Transcript signatures in pooled tumor types. (XLS 34 kb) Additional file 8: Predictive signatures based on gene expression and splicing events. (XLS 278 kb) Additional file 9: Models in ER+/ER– breast and MITF+/MITF– melanoma tumors. (XLS 177 kb)
