# Supplementary material for: The prognostic potential of alternative transcript isoforms across human tumors
Source: Genome Med. 2016 Aug 17;8:85. doi: 10.1186/s13073-016-0339-3 (PMC4989457; doi:10.1186/s13073-016-0339-3)
Supplement: Additional file 2: — This additional file contains the supplementary figures cited in the paper. (PDF 1828 kb) [file 13073_2016_339_MOESM2_ESM.pdf]

Additional file 2.

This additional file contains the supplementary figures cited in the paper.

Figure S1. **(a)** Information-based feature selection methods provide a robust and conservative measure of the discriminant power of features. Left panels show plots comparing the information gain (IG) (upper panel), gain ratio (GR) (middle panel) and symmetrical uncertainty (SU) (lower panel) (y axes) with the Wilcoxon-test p-value after multiple-testing correction using Benjamini-Hochberg method (x axes) for the distribution of PSI values for two patient subgroups. In this case, the data corresponds to the comparison between ER+ and ER- breast tumor samples, subsampling 20 patients per group. Each dot corresponds to one isoform in each of the subsamples. Right panels: in red we show the distributions of IG (upper panel), GR (middle panel) and SU (lower panel) values for the comparison of a transcript from MAP3K7 between ER+ and ER- samples with using 100 subsamples of size 20. In blue we show the same values in the comparison between the groups after shuffling the labels. **(b)** Enriched hallmarks (y axis) for each stage class, metastasis (M), tumor size (T), lymph-node involvement (N) and overall staging (S), in each tumor type (x axis), using all discriminant isoforms found in each case. Only significant cases (corrected Fisher test p-value < 0.05) are shown. **(c)** Comparison of the ΔPSI values for the discriminant isoforms between metastatic and non-metastatic SKMC samples (x axis) with the ΔPSI values from the comparison of the metastatic (SKMel147) and non-metastatic (Mel505) melanoma cells (y axis). We indicate in blue or red those isoforms with the same or opposite change direction, respectively. Dark and light colors indicate |ΔPSI|>0.1 and |ΔPSI|<0.1, respectively. The correlation (Pearson R) is given for isoforms in dark blue. **(d)** Accuracy of the models in terms of the areas under the precision-recall curves (PRC) (y axis) for the late-stage classes (i.e. precision is measured as the proportion of predicted late stage samples that are correctly predicted). The bars show the minimum, mean and maximum values of the area of the precision-recall curves. Some models are absent due to lack of sufficient samples (Table 1).

Figure S2. (a) PSI distributions of some of the transcript isoforms in the derived predictive signatures. From left to right, *PAX6* isoform in the KIRP T-model for KIRP (Wilcoxon test p-value = 2.695e-06), *MKNK1* isoform in the KIRP N-model (Wilcoxon test p-value = 0.0004), *TM6SF1* isoform in the SKCM M-model (Wilcoxon test p-value = 1.813e-05), *PRDM16* isoform (Wilcoxon test p-value = 0.0001) and *PTKB* isoform (Wilcoxon test p-value = 0.005) in BRCA S-model. The y-axis indicates the PSI value in each sample separated according to early and late stages (x-axis). **(b)** Left panel: XY-plot of the PSI values (y axis) of the *ENAH* isoform that appears in the T-models of KIRP and COAD, and the stromal score (x axis), across all COAD tumor samples. Pearson correlation with stromal score R=-0.59 and with immune score R=-0.41. Right panel: PSI distribution of the same *ENAH* isoform in early and late T-stages in KIRP and COAD (Wilcoxon test p-value < 0.001).

Figure S3. (a) Accuracies of the transcript isoform models (I) compared to the gene (G) and mixed (M) combining isoform and gene information. Accuracies are given as boxplots for the distribution of AUC values (y axis) from a 10-fold cross-validation for each tumor type (x axis) for the metastasis (M), overall stage (S), lymph node invasion (N) and tumor size (T) models (panels from upper left to lower right). Tumors for which stage data was missing are not shown (see Table 1 of the manuscript). **(b)** and **(c)**: Survival (Kaplan-Meyer) plot associated to the blind test for N and S stage, respectively using the mixed model combining isoform and gene information. The plot indicates the survival percentage (y axis) versus survival in months (x axis) based on the predicted stage on the unannotated samples using the classifier for each corresponding tumor type. The p-value in each plot corresponds to the Cox regression between the two groups and HR indicates the hazards ratio

Figure S4. (a) Comparison of the ΔPSI values for the discriminant isoforms between ER+ and ER- samples (x axis) with the ΔPSI values from the comparison of the control and knockdown of *ESR1* in MCF7 cells (y axis). We indicate in blue or red those isoforms with the same or opposite change direction, respectively. Dark and

light colors indicate |ΔPSI|>0.1 and |ΔPSI|<0.1, respectively. The correlation (Pearson R) is given for isoforms in dark blue. From the 2337 transcript isoforms with expression in the MCF7 experiments, 1123 (48%) show PSI changes in the same direction and 328 of them with |ΔPSI| > 0.1. **(b)** PSI distribution of the *MAP3K7* isoform that changes significantly between the ER+ and ER- BRCA sets (Wilcoxon test p-value < 2.2E-16). Plots **(c)** and **(d)** show the survival (Kaplan-Meyer) curves for the ER- samples according to early and late N and S stages, respectively. The p-value in each plot corresponds to the Cox regression between the two groups and HR indicates the hazards ratio. **(e)** Enriched cancer hallmarks for the set of discriminant isoforms between ER+ and ER- subsets (ER+_ER-) and for the set of isoforms separating early and late stages in ER- (ER-). For this latter comparison isoforms associated to N, T and S stages were combined into early and late subgroups. **(f)** Accuracy of the models in terms of the areas of the precision-recall curves (PRC) (y axis) for the comparison between ER+ and ER- subgroups and for the comparison of early vs late stage classes in each subtype, ER+ and ER-, for N, S and T annotation. The precision is measured as the proportion of predicted late stage samples or ER+ samples that are correctly predicted. The bars show the minimum, mean and maximum values of the area of the precision-recall curves.

Figure S5. (a) Comparison of the ΔPSI values for the discriminant isoforms between MITF+ and MITF- melanoma tissue samples (x axis) with the ΔPSIs obtained from the comparison of the control and knockdown of *MITF* in Mel505 cells (y axis). We indicate in blue or red those isoforms with the same or opposite change direction, respectively. Dark and light colors indicate |ΔPSI|>0.1 and |ΔPSI|<0.1, respectively. The correlation (Pearson R) is given for isoforms in dark blue. From the total of 2279 discriminant isoforms for which we found expression in the cell lines, 1050 (46%) show a ΔPSI change in the same direction, with 865 of them having |ΔPSI|>0.1. **(b)** Enriched cancer hallmarks (y axis) (corrected Fisher test p-value < 0.05) using the discriminant isoforms in the comparison MITF- vs MITF+ and comparing low and high survival subgroup of patients within each subtype MITF+ or MITF-. Enriched hallmarks were the same using the top and bottom 10% or 25% samples according to *MITF* expression to define the subtypes. **(c)** PSI distributions of the *TPM1* isoform (left panel) and *RAB27A* isoform (right panel) that separate the two melanoma

subtypes, MITF+ and MITF- (Wilcoxon test p-values = 5.293e-9 and 4.86e-12, respectively). The plots indicate the PSI values (y-axis) for the isoforms in MITF+ and MITF- samples (x-axis). **(d)** Genomic locus for RAB27A indicating the annotated isoforms; uc002acr.2 decreases PSI in MITF+, whereas uc002acp.2 increases PSI in MITF+. **(e)** Accuracy given in terms of the areas under the precision-recall curves (PRC) (y axis) from a 10-fold cross-validation for (from left to right in the x axis) the survival model for MITF+, MITF- as well as for the separation between MITF+ and MITF- subgroups using 25% (Q1 vs Q4) or 10% (D1 vs D10) of the top and bottom samples in the ranking of *MITF* expression The bars show the minimum, mean and maximum values of the area of the precision-recall curves.

Figure S1

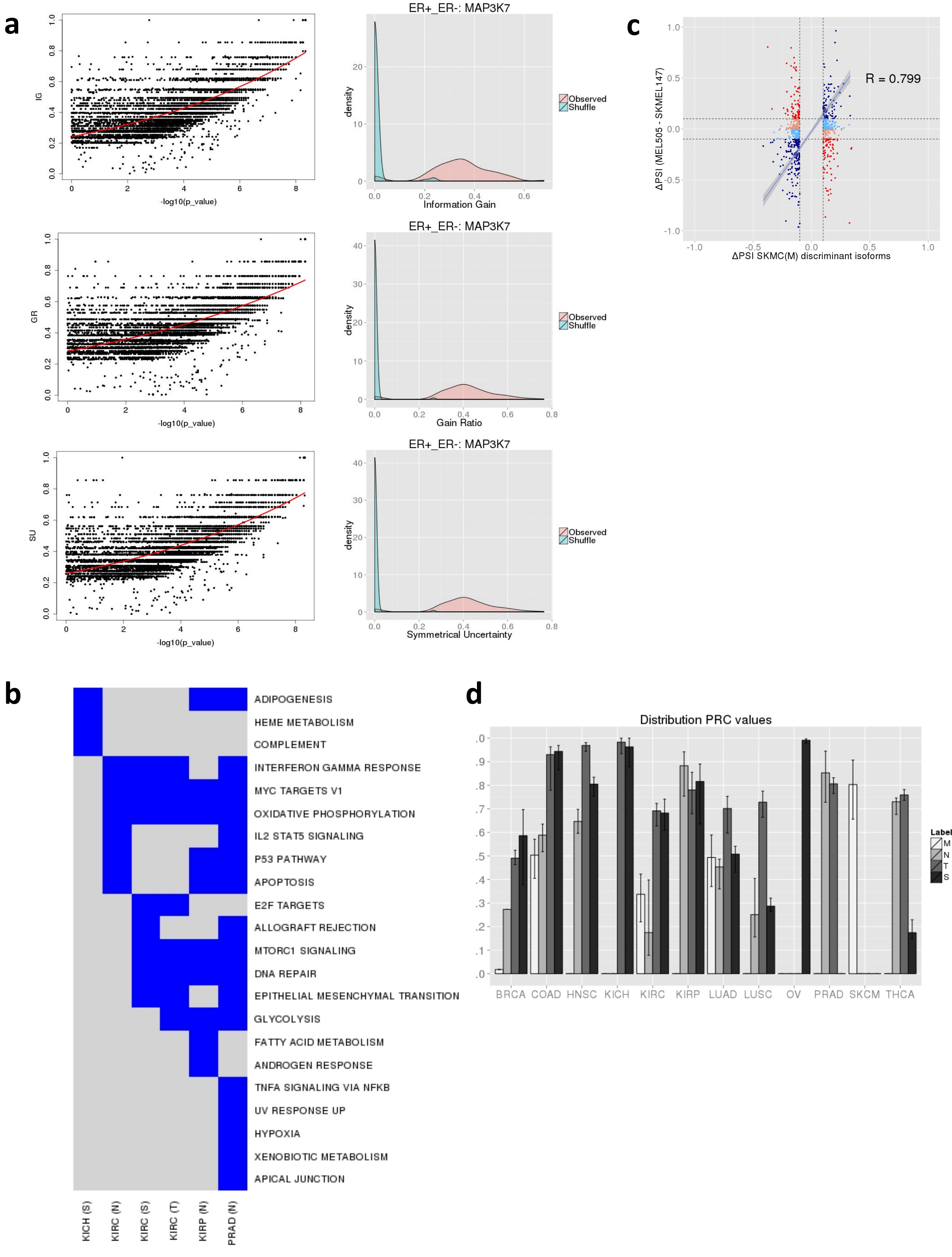

a
IG
-log10(p_value)
ER+_ER-: MAP3K7
density
Information Gain
Observed
Shuffle
GR
Gain Ratio
SU
Symmetrical Uncertainty
c
R = 0.799
ΔPSI (MEL505 - SKMEL147)
ΔPSI SKMC(M) discriminant isoforms
b
ADIPOGENESIS
HEME METABOLISM
COMPLEMENT
INTERFERON GAMMA RESPONSE
MYC TARGETS V1
OXIDATIVE PHOSPHORYLATION
IL2 STAT5 SIGNALING
P53 PATHWAY
APOPTOSIS
E2F TARGETS
ALLOGRAFT REJECTION
MTORC1 SIGNALING
DNA REPAIR
EPITHELIAL MESENCHYMAL TRANSITION
GLYCOLYSIS
FATTY ACID METABOLISM
ANDROGEN RESPONSE
TNFA SIGNALING VIA NFKB
UV RESPONSE UP
HYPOXIA
XENOBIOTIC METABOLISM
APICAL JUNCTION
KICH (S)
KIRC (N)
KIRC (S)
KIRC (T)
KIRP (N)
PRAD (N)
d
Distribution PRC values
Label
M
N
T
S
BRCA
COAD
HNSC
KICH
KIRC
KIRP
LUAD
LUSC
OV
PRAD
SKCM
THCA

Figure S2

a

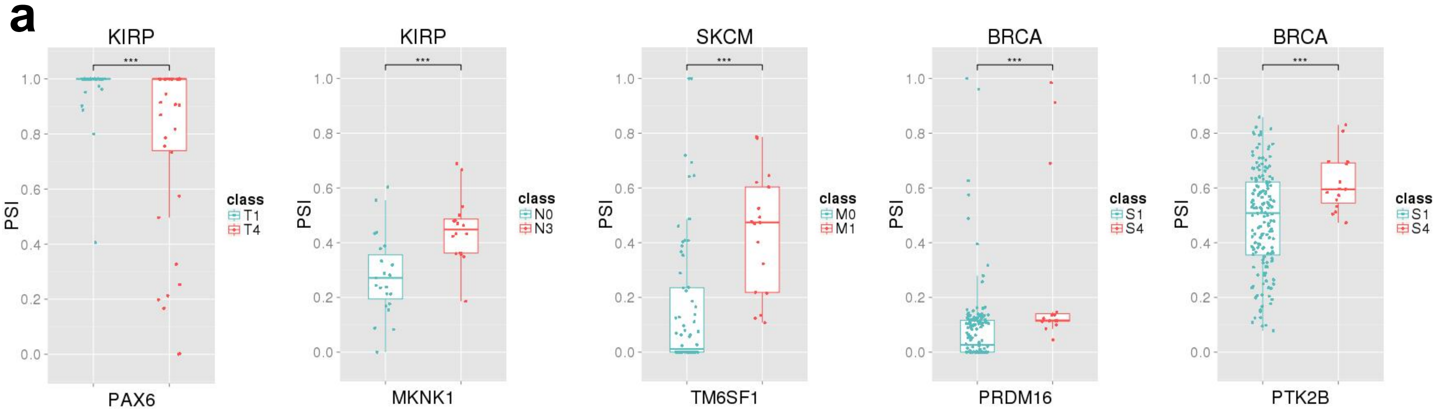
KIRP
KIRP
SKCM
BRCA
BRCA
PSI
class
T1
T4
N0
N3
M0
M1
S1
S4
PAX6
MKNK1
TM6SF1
PRDM16
PTK2B

b

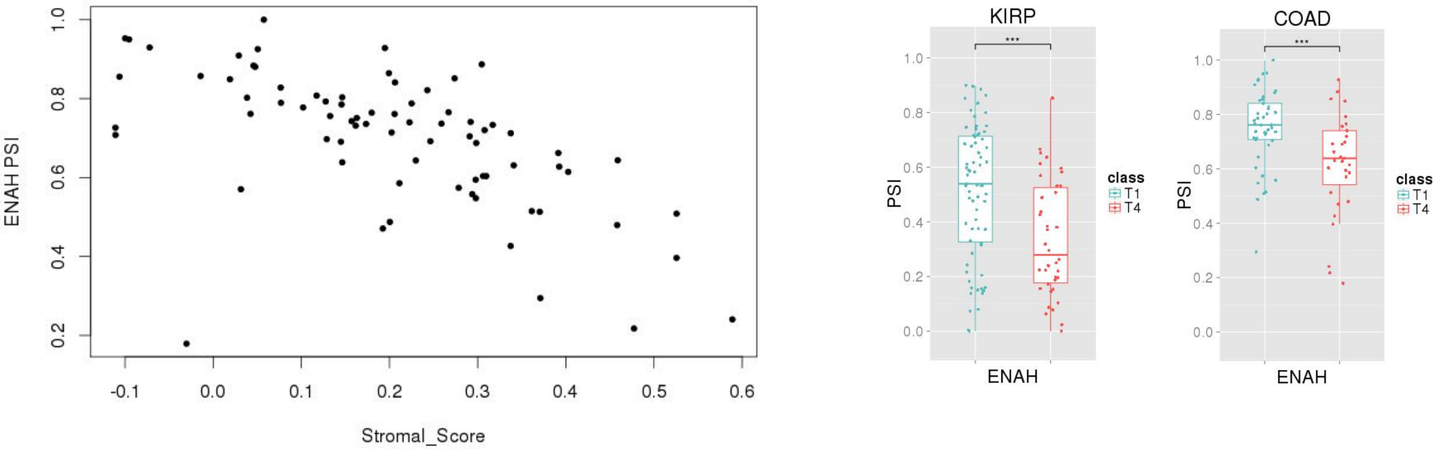
ENAH PSI
Stromal_Score
KIRP
COAD
PSI
class
T1
T4
ENAH

Figure S3

a

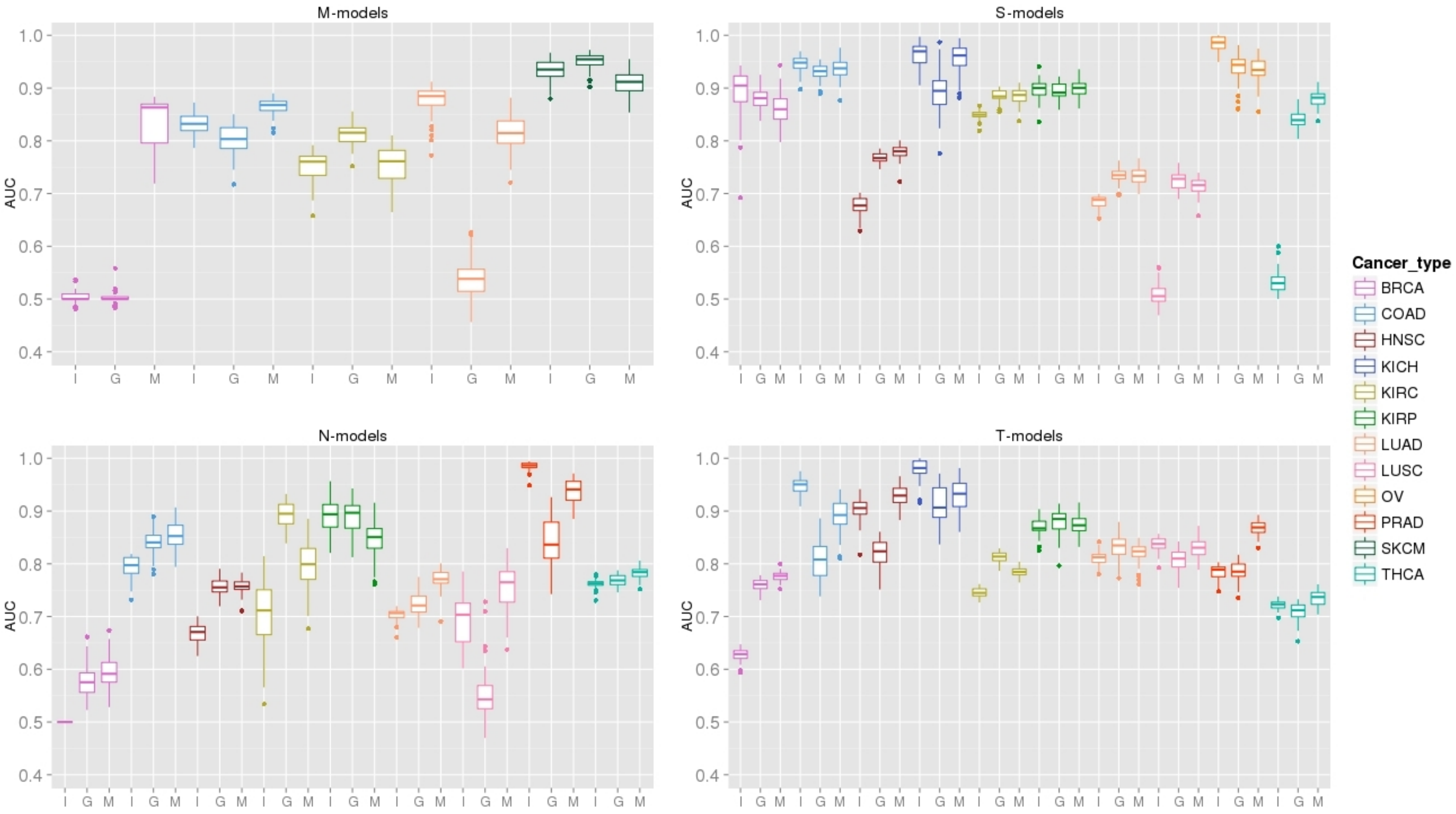

b

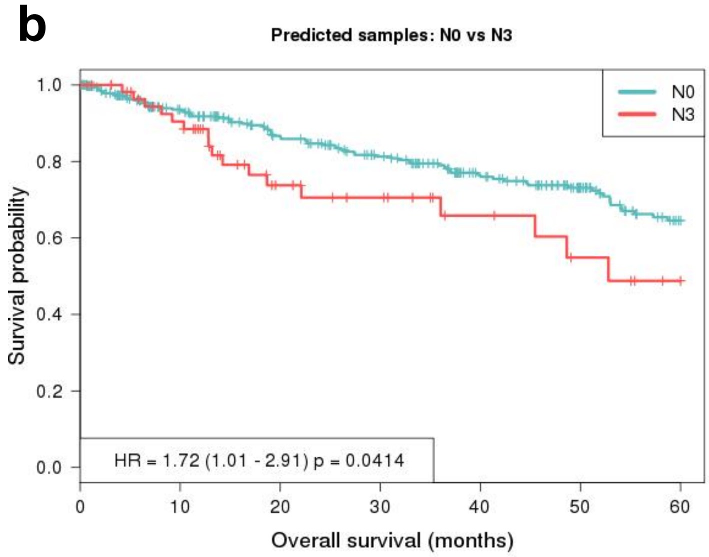

c

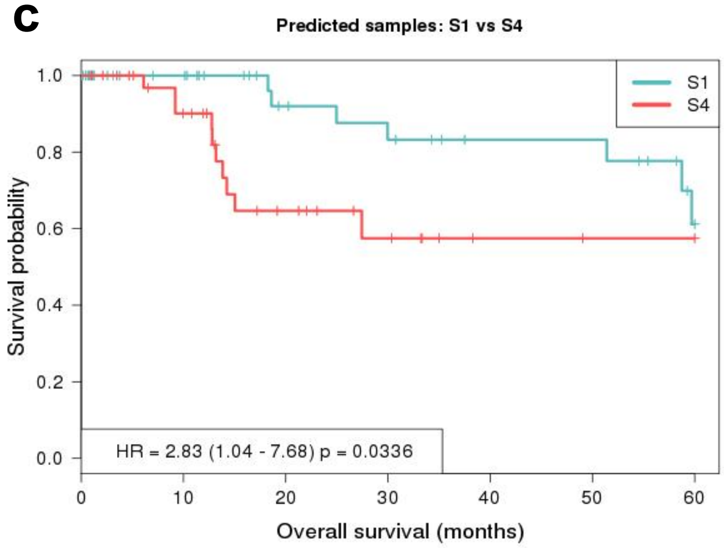

Figure S4

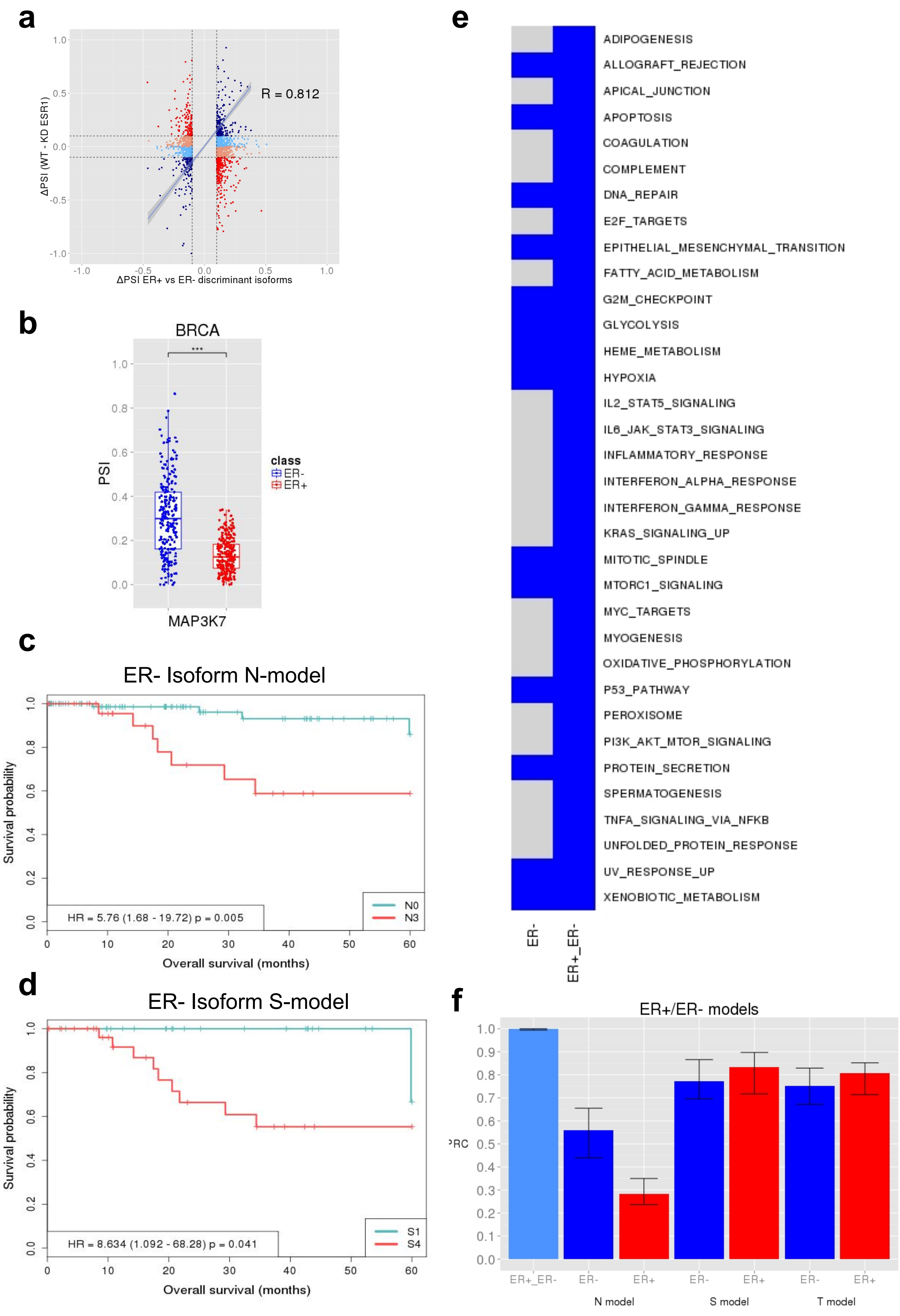

a
ΔPSI (WT - KD ESR1)
R = 0.812
ΔPSI ER+ vs ER- discriminant isoforms
b
BRCA
PSI
class
ER-
ER+
MAP3K7
c
ER- Isoform N-model
Survival probability
HR = 5.76 (1.68 - 19.72) p = 0.005
N0
N3
Overall survival (months)
d
ER- Isoform S-model
Survival probability
HR = 8.634 (1.092 - 68.28) p = 0.041
S1
S4
Overall survival (months)
e
ADIPOGENESIS
ALLOGRAFT_REJECTION
APICAL_JUNCTION
APOPTOSIS
COAGULATION
COMPLEMENT
DNA_REPAIR
E2F_TARGETS
EPITHELIAL_MESENCHYMAL_TRANSITION
FATTY_ACID_METABOLISM
G2M_CHECKPOINT
GLYCOLYSIS
HEME_METABOLISM
HYPOXIA
IL2_STAT5_SIGNALING
IL6_JAK_STAT3_SIGNALING
INFLAMMATORY_RESPONSE
INTERFERON_ALPHA_RESPONSE
INTERFERON_GAMMA_RESPONSE
KRAS_SIGNALING_UP
MITOTIC_SPINDLE
MTORC1_SIGNALING
MYC_TARGETS
MYOGENESIS
OXIDATIVE_PHOSPHORYLATION
P53_PATHWAY
PEROXISOME
PI3K_AKT_MTOR_SIGNALING
PROTEIN_SECRETION
SPERMATOGENESIS
TNFA_SIGNALING_VIA_NFKB
UNFOLDED_PROTEIN_RESPONSE
UV_RESPONSE_UP
XENOBIOTIC_METABOLISM
ER-
ER+_ER-
f
ER+/ER- models
ER+_ER-
ER-
ER+
N model
S model
T model

Figure S5

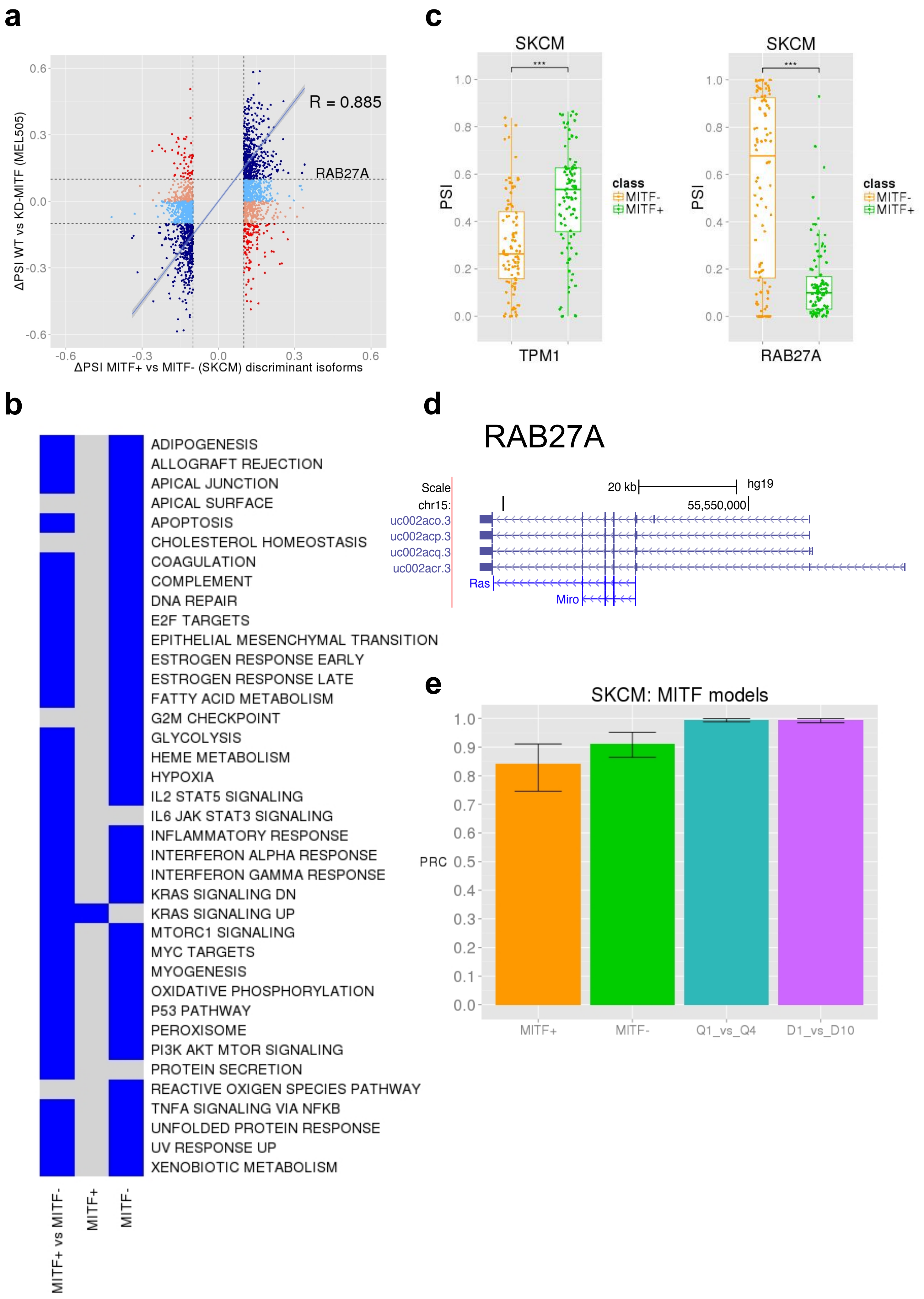
a
R = 0.885
RAB27A
ΔPSI WT vs KD-MITF (MEL505)
ΔPSI MITF+ vs MITF- (SKCM) discriminant isoforms
0.6
0.3
0.0
-0.3
-0.6
c
SKCM
SKCM
***
***
PSI
1.0
0.8
0.6
0.4
0.2
0.0
class
MITF-
MITF+
TPM1
RAB27A
b
ADIPOGENESIS
ALLOGRAFT REJECTION
APICAL JUNCTION
APICAL SURFACE
APOPTOSIS
CHOLESTEROL HOMEOSTASIS
COAGULATION
COMPLEMENT
DNA REPAIR
E2F TARGETS
EPITHELIAL MESENCHYMAL TRANSITION
ESTROGEN RESPONSE EARLY
ESTROGEN RESPONSE LATE
FATTY ACID METABOLISM
G2M CHECKPOINT
GLYCOLYSIS
HEME METABOLISM
HYPOXIA
IL2 STAT5 SIGNALING
IL6 JAK STAT3 SIGNALING
INFLAMMATORY RESPONSE
INTERFERON ALPHA RESPONSE
INTERFERON GAMMA RESPONSE
KRAS SIGNALING DN
KRAS SIGNALING UP
MTORC1 SIGNALING
MYC TARGETS
MYOGENESIS
OXIDATIVE PHOSPHORYLATION
P53 PATHWAY
PEROXISOME
PI3K AKT MTOR SIGNALING
PROTEIN SECRETION
REACTIVE OXIGEN SPECIES PATHWAY
TNFA SIGNALING VIA NFKB
UNFOLDED PROTEIN RESPONSE
UV RESPONSE UP
XENOBIOTIC METABOLISM
MITF+ vs MITF-
MITF+
MITF-
d
RAB27A
Scale
chr15:
20 kb
hg19
55,550,000
uc002aco.3
uc002acp.3
uc002acq.3
uc002acr.3
Ras
Miro
e
SKCM: MITF models
PRC
1.0
0.9
0.8
0.7
0.6
0.5
0.4
0.3
0.2
0.1
0.0
MITF+
MITF-
Q1_vs_Q4
D1_vs_D10
